# Ibuprofen in a Lipid Bilayer: Nanoscale Spatial Arrangement

**DOI:** 10.3390/membranes12111077

**Published:** 2022-10-30

**Authors:** Anna S. Kashnik, Denis S. Baranov, Sergei A. Dzuba

**Affiliations:** Voevodsky Institute of Chemical Kinetics and Combustion, SB RAS, 630090 Novosibirsk, Russia

**Keywords:** non-steroidal anti-inflammatory drug, NSAID, lipid bilayer, clustering, spin-label EPR, DEER, PELDOR

## Abstract

Ibuprofen is a non-steroidal anti-inflammatory drug (NSAID) with analgesic and antipyretic effects. Understanding the molecular mechanisms of drug interaction with cell membranes is important to improving drug delivery, uptake by cells, possible side effects, etc. Double electron-electron resonance spectroscopy (DEER, also known as PELDOR) provides information on the nanoscale spatial arrangement of spin-labeled molecules. Here, DEER was applied to study (mono-)spin-labeled ibuprofen (ibuprofen-SL) in a bilayer of palmitoyl-oleoyl-sn-glycerophosphocholine (POPC). The results obtained show that the ibuprofen-SL molecules are located within a plane in each bilayer leaflet. At their low molar concentration in the bilayer χ, the found surface concentration of ibuprofen-SL is two times higher than χ, which can be explained by alternative assembling in the two leaflets of the bilayer. When χ > 2 mol%, these assemblies merge. The findings shed new light on the nanoscale spatial arrangement of ibuprofen in biological membranes.

## 1. Introduction

Ibuprofen is a non-steroidal anti-inflammatory drug (NSAID) widely used for analgetic and antipyretic effects [1,2,3]. It is known that it blocks the synthesis of prostaglandins due to the inhibition of the membrane enzyme cyclooxygenase, reduces the parallel side effects in the gastrointestinal tract [4], and slows down neurodegenerative diseases [5,6,7]. Additionally, ibuprofen can be effective as an anticancer agent [8,9,10,11]. Ibuprofen-containing drugs are known to be effective in combination with other drugs [12,13,14]. Ibuprofen was studied for the purposes of increasing its bioavailability [15], for solving the problems of drug delivery [15,16], and many other purposes. 

Elucidation of the molecular mechanisms of interaction of drugs with cell membranes is important to improve their delivery and uptake by cells, thereby reducing possible side effects. Various experimental approaches were used to conduct research in this direction [16,17,18,19,20,21,22,23,24]. These studies discussed, among other issues, the nanoscopic structure and dynamics of ibuprofen-containing membranes. 

Spin-label electron paramagnetic resonance (EPR) spectroscopy can also be used to investigate the ibuprofen-membrane interaction [25,26,27]. The results obtained with conventional continuous wave (CW) EPR showed that this poorly water-soluble drug is incorporated into the model lipid membranes by binding through non-specific interactions at the polar/apolar interface [26]. It was also found that ibuprofen decreases the molecular packing of the polar heads, leaving untouched the chain flexibility in the liquid-crystalline phase. In [27], spin-probe EPR was used to probe the interaction of spin-labeled ibuprofen (ibuprofen-SL) with a model lipid bilayer. Electron spin echo envelope modulation (ESEEM) spectroscopy was employed in this work to determine the location of ibuprofen-SL in the bilayer; the found immersion depth into the membrane was in agreement with the literature data on Molecular Dynamics (MD) simulation in a lipid bilayer [26,28,29] for the (unlabeled) ibuprofen molecule. 

Here we study the interaction of ibuprofen with the membrane using ibuprofen-SL and pulse EPR in the version of Double Electron-Electron Resonance (DEER, also known as PELDOR) [30,31,32,33], which employs a series of microwave pulses of the EPR resonance frequencies. An important advantage of DEER spectroscopy is that it probes the spin-spin dipole-dipolar interaction between spin labels separated by nanoscale distances ranging between 1.5 and ~8 nm. 

DEER spectroscopy was originally invented [30] as a three-pulse technique using two independent home-built pulse EPR spectrometers. Then it was improved [31] with a four-pulse measurement scheme to eliminate the dead-time problem that occurs with a (single) commercial spectrometer. Next, a modification of the three-pulse measurement scheme was proposed [34], which also removed this problem for a single spectrometer. In this work, we use this measurement scheme since the three-pulse signal is larger than the four-pulse one [34]. 

So far, DEER spectroscopy has been applied mainly to double-spin-labeled biomolecules in order to study their conformation. It has recently been shown that DEER, as applied to mono-spin-labeled molecules, can also reveal the features of their nanoscale spatial arrangement [35,36]. Here we also use mono-spin-labeled ibuprofen.

## 2. Theoretical Background

DEER spectroscopy [30,31,32,33] is based on the electron spin echo (ESE) phenomenon. An ESE signal appears when a spin system in a magnetic field is subjected to microwave pulses of the EPR resonance frequency. For a pair of spins, A and B, in which the spin A is excited by two or three echo-forming pulses at the microwave frequency ν_A_, and the spin B is excited by a pumping pulse at the microwave frequency ν_B_, the A spin echo signal is determined as [30,31,32,33]:

(1) $$e_{A}(t)=1-p_{B}+p_{B}\cos\frac{g^{2}\mu_{B}^{2}}{\hbar r_{AB}^{3}}(1-3\cos^{2}\theta_{AB})t$$

here *g* is the *g*-factor, $\mu_{B}^{}$ is the Bohr magneton, $\theta_{AB}$ is the angle between vector **r***_AB_* and the external magnetic field **B**_0_, *p_B_* < 1 is a dimensionless parameter determining the efficiency of the pumping excitation, and *t* is the time delay for the pumping pulse (see Section 3). 

For a macroscopic system of mono-spin-labeled molecules, Equation (1) is to be multiplied over all spin pairs, which produces the DEER signal:

(2) $$V(t)=\prod_{j>i}\left[1-p_{B}+p_{B}\cos\frac{g^{2}\mu_{B}^{2}}{\hbar r_{ij}^{3}}(1-3\cos^{2}\theta_{ij})t\right]$$

To perform further calculations, all *i* spins in this multiplication without loss of generality may be placed at the origin of coordinates. For a random three-dimensional (3-D) spatial distribution, the probability for the given spin to be located at a position **r** in a small elementary space element *d***r** is equal to the ratio *d***r**/*V*, where *V* is the macroscopic volume. Then Equation (2) is transformed into:

(3) $$V_{3D}(t)={\left[1-\frac{dr}{V}p_{B}(1-\cos\frac{g^{2}\mu_{B}^{2}}{\hbar r_{}^{3}}(1-3\cos^{2}\theta_{})t)\right]}^{N}$$

where *N* is the total number of spins, *N* = *CV* where *C* is the spin concentration. Since *N* and *V* are macroscopically large, they can be considered as tending to infinity, keeping the ratio *N/V = C* constant [37]. Then one obtains:

(4) $$V_{3D}(t)\underset{N,V\to \infty }{\to }\exp(-Cp_{B}\int dr[1-\cos\frac{g^{2}\mu_{B}^{2}}{\hbar r_{}^{3}}(1-3\cos^{2}\theta )t])$$

Integration results in [30,38]:

(5) $$V_{3D}(t)=\exp(-\frac{8\pi^{2}}{9\sqrt{3}}\frac{g^{2}\mu_{B}^{2}}{\hbar }Cp_{B}t)$$

Note that *C* is taken here in the cm^−3^ units.

For lipid bilayers, the spatial distribution of spin labels is expected to be closer to the two-dimensional (2-D) one. When spin labels are located in a single plane, one can obtain that Equation (2) is transformed into the dependence in the form of $\exp(-const\cdot \sigma t^{2/3})$ [39], where *σ* is the surface spin concentration. For the 2-D case, one can perform similar calculations as for the 3-D case, with an intermediate parameter of surface area *S* instead of volume *V*. Averaging over all angles *α* determining the orientation of the magnetic field respectively to the membrane normal then gives:

(6) $$V_{2D}(t)=\exp\left(-\sigma p_{B}\frac{1}{2}\int_{0}^{\pi }\sin\alpha d\alpha \int_{0}^{2\pi }d\varphi \int_{0}^{\infty }rdr[1-\cos(\frac{g^{2}\mu_{B}^{2}t}{r^{3}}(1-3\sin^{2}\alpha \cos^{2}\varphi ))]\right)$$

Here, the angle *ϕ* determines the orientation of the vector **r** in the membrane plane. The surface density *σ* is taken here in the cm^−2^ units. By proper substitution of variables, one obtains that Equation (6) may be reduced to:

(7) $$V_{2D}(t)=\exp\left(-\sigma p_{B}{\left(g^{2}\mu_{B}^{2}t\right)}^{2/3}I_{1}I_{2}\right)$$

where the two integrals;

(8) $$\begin{array}{l} I_{1}=\int_{0}^{1}dx\int_{0}^{2\pi }d\varphi |1-\cos(1-3(1-x^{2})\cos^{2}\varphi ){|}^{2/3}≅5.037,\\ I_{2}=\frac{1}{3}\int_{0}^{\infty }\frac{1-\cos y}{y^{5/3}}≅0.670 \end{array}$$

are calculated numerically. Then we obtained:

(9) $$V_{2D}(t)=\exp(-3.37\sigma p_{B}{\left(\frac{g^{2}\mu_{B}^{2}}{\hbar }t\right)}^{\frac{2}{3}})$$

This result may also be applied to the case of inhomogeneous spin distribution, for which the concentrations *C* in Equation (5) and *σ* in Equation (9) are to be replaced by *C_local_* and *σ_local_*, correspondingly. Note that typical distances studied in DEER, $r\;\sim \;{\left(g^{2}\mu_{B}^{2}t/\hbar \right)}^{1/3}$ (see Equation (1)) are of several nm for *t* ~ 10^-6^ s, which is typical for molecular solids. Simulations performed in [40] have shown that this replacement can be used for an inhomogeneity size larger than 20 nm, containing a number of spins > 10^2^. 

It should be noted that in [40], Equation (6) was numerically calculated for spin labels locating within a ring of the 200 nm radius, with the restriction that they cannot be closer than 0.5 nm apart, which resulted in a slightly smaller numerical coefficient in Equation (9) (3.21 instead of 3.37 in this work).

## 3. Experimental

### 3.1. Materials and Sample Preparation

**Figure 1:**
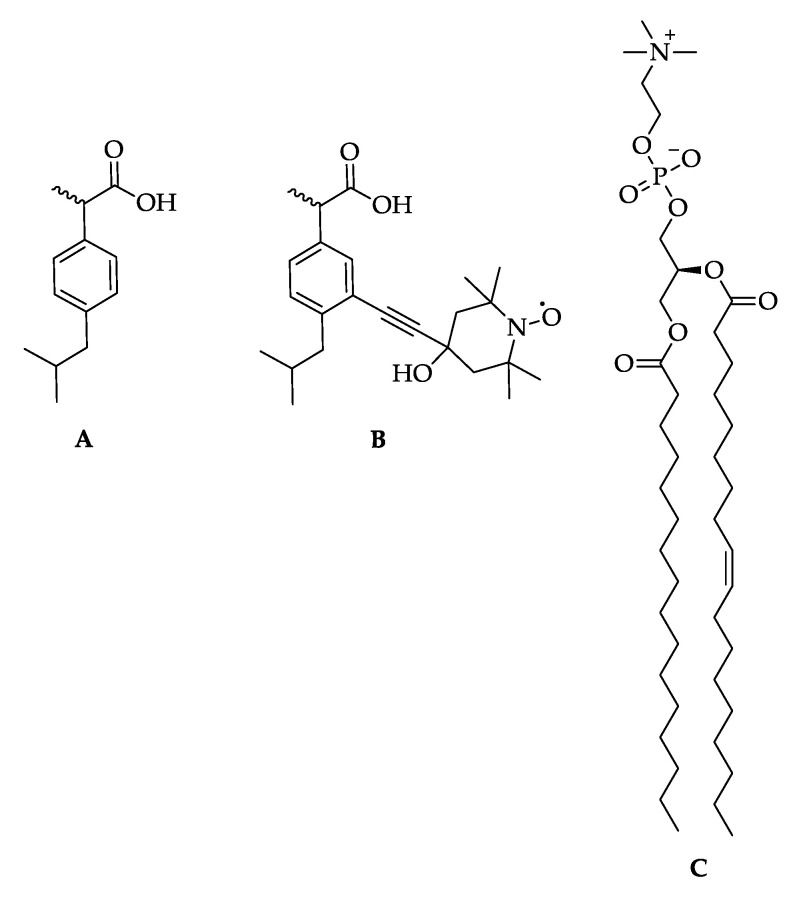
Chemical structures: (**A**) ibuprofen, (**B**) spin-labeled ibuprofen (ibuprofen-SL), and (**C**) zwitterionic lipid POPC.

The model membrane was a 1-palmitoyl-2-oleoyl-sn-glycero-3-phosphocholine (POPC) bilayer. POPC was obtained from Avanti Polar Lipids (Birmingham, AL, USA). Ibuprofen-SL was synthesized in the same manner as previously described [27]. The chemical structures of ibuprofen, ibuprofen-SL and POPC are shown in Figure 1.

Ibuprofen-SL and POPC were separately dissolved in chloroform, then the solutions were mixed in the required molar proportions. The solvent was then removed in the nitrogen stream; the mixture was subsequently stored under vacuum for 4 h. Then an excess of phosphate-buffered saline (pH 7.0) was added in a proportion of 10:1. The sample was stirred and then stored for 2 h. Throughout this procedure, multilamellar vesicles (MLVs) are formed [41]. Then the sample was centrifuged to remove the excess solvent. The prepared sample was put into an EPR glass tube of 3 mm o.d. and studied either at room temperature, or at 200 K, or at 80 K. In the two latter cases, the samples were quickly frozen by immersion into liquid nitrogen.

### 3.2. EPR Measurements

An X-band Bruker ELEXSYS E580 EPR spectrometer was employed in all measurements. CW EPR spectra were obtained using a Bruker ER 4118X-MD5 dielectric resonator. The modulation amplitude was 0.01 mT, the output microwave power was 200 mW, the microwave attenuation was −25 dB, the sweep time and the spectrometer time constant were 10.49 s and 20.48 ms, respectively. 

In pulsed EPR studies, the spectrometer was equipped with a split-ring Bruker ER 4118 X-MS-3 resonator. A three-pulse DEER measuring scheme was used (*π*/2)*_νA_ − t* − *π_νB_* − (*t − τ*) − *π_νA_ − τ − echo_νA_*, where the subscripts denote the microwave frequencies. The lengths of the first and second pulses applied at the frequency *ν_A_* were 16 and 32 ns, respectively. The pumping pulse was of 36 ns length, and its amplitude was adjusted to provide a *π* turning angle. The time delay *τ* was 700 ns. The pumping pulse delay *t* was scanned with a step of 4 ns, starting from *t* = −176 ns before the first detection pulse. In the applied spectrometer magnetic field, the frequency *ν_B_* corresponded to the excitation of the maximum of the echo-detected EPR spectrum, while detection was performed at the frequency *ν_A_* corresponding to its high-field shoulder; therefore the difference *ν_B_* – *ν_A_* was 85 MHz. The distortion of the DEER signal during the passage of the pump pulse through the detecting pulses was corrected by comparison with the "idle" excitation [34], which ensured the absence of dead time in the experiment. The zero-time delay *t* was established in a calibration experiment described in [42]. The sample temperature in DEER experiments was kept near 80 K.

In measurements at a reduced temperature, the resonator was placed into an Oxford Instruments CF-935 cryostat and cooled with a stream of cold nitrogen gas. The temperature was stabilized by a Bruker ER4131VT temperature controller. 

## 4. Results and Discussion

**Figure 2:**
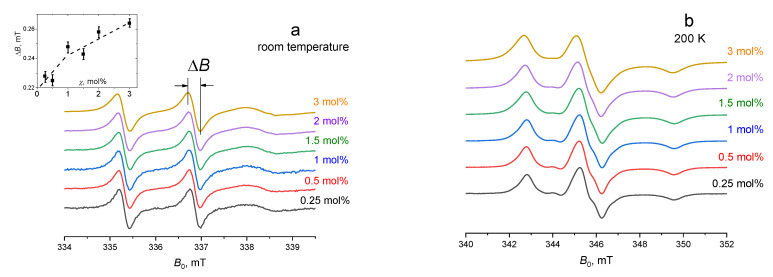
EPR spectra for different concentrations of ibuprofen-SL in POPC bilayer taken (**a**) at room temperature and (**b**) at 200 K. The insert shows the concentration dependence of the central component width at room temperature. The dashed line is drawn to guide the eye.

The CW EPR spectra obtained for various molar concentrations *χ* of ibuprofen-SL in POPC bilayers are shown in Figure 2a for room temperature and in Figure 2b for 200 K. At room temperature, the spectra are similar to those published previously in [27], where it was shown that these spectra indicate direct binding of ibuprofen to the lipid bilayer. As it is seen from the insert to Figure 2a, the spectral linewidth slightly increases with concentration. The increase can be either due to spin-spin interactions between spin labels (spin-exchange or magnetic dipole-dipole combinations) [43] or due to the slowing down of the motion [43]. The effect is not large, with the linewidth increasing by 15 % only, but nevertheless, it is above the experimental uncertainty. At 200 K (Figure 2b), the spectra show that the spin labels are immobilized [43], with a slight concentration dependence of the line shape. 

**Figure 3:**
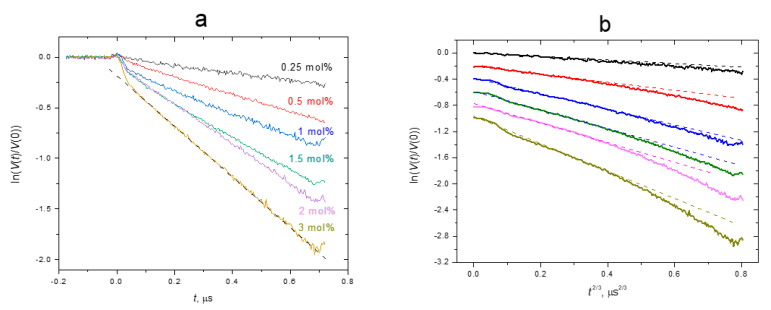
Semilogarithmic plot of three-pulse DEER time traces for ibuprofen-SL in the POPC bilayer taken for different molar concentrations *χ*. (**a**) The data are plotted vs. *t*. (**b**) The data are plotted vs. *t*^2/3^ and shifted vertically for convenience. Straight dashed lines present linear approximations (see text).

The obtained DEER time traces are shown in Figure 3 as semilogarithmic plots, with two different abscissa axes: *t* in Figure 3a and *t*^2/3^ in Figure 3b. One can see in Figure 3a the fast initial decay of the signal occurring within the time interval 0 < *t* < 40 ns; this decay is then substituted by a slow linear dependence. At first glance, the linearity is consistent with the theoretical Equation (5) (neglecting for a while the initial fast decay). However, this equation fails to provide a quantitative description of the experimental data. Indeed, the *C_local_* value cannot be smaller than the *χC*_lipids_ where *C*_lipids_ is the volume concentration of the lipids in the bilayer. The latter may be assessed from data in [44] for the POPC bilayer, as 1/*V_L_* ≈ 8 × 10^20^ cm^−3^, where *V_L_* is the volume per lipid. The *p_B_* value for ibuprofen-SL was previously reported by us [27] as 0.22. Then one obtains from the data in Figure 3a for the 3 mol% sample that Equation (5) results in the ratio *C_local_*/*C*_lipids_ ≈ 0.9 mol%, which is several times smaller. Thus, we conclude that the 3-D distribution cannot be used to describe the obtained DEER experimental data.

Therefore, we will turn to the 2-D model of the spatial distribution of ibuprofen, which, of course, seems to be more suitable for membranes. Indeed, as was stated above, both experimental [27] and MD simulation [26,28,29] data show that ibuprofen molecules are located near the membrane surface. This is an obvious consequence of the amphiphilic nature of its molecular structure (see Figure 1A), with a hydrophilic carboxyl group–COOH and a hydrophobic residue consisting of a phenyl ring and an isobutyl tail. Additionally, it was concluded [27] that the presence of a spin label (with the hydrophilic OH group) does not influence the immersion depth of the ibuprofen molecule. 

To compare the DEER time traces with the theoretical Equation (9) for the 2-D distribution, the experimental data are plotted in Figure 3b vs. *t*^2/3^. First, we note from these data that the fast initial fast decay seen in Figure 3a disappears. However, the experimental curves here are non-linear. But this nonlinearity in Figure 3b is rather small and can be easily attributed to the imperfection of the model used. The imperfection may appear because of the inter-plane interaction between spin labels in the two opposite leaflets. Indeed, non-linearity was demonstrated in simulations performed in [36] (see in this work Figure S7 in the Supporting Information). Simulations [36] for inter-plane interaction predict zero slope at low time delays for the time dependence of the DEER signal, so the linear approximation of the initial slope in Figure 3b can be directly used to apply Equation (9)—thus neglecting inter-plane interaction. These linear approximations are shown in Figure 3b by dashed straight lines.

**Figure 4:**
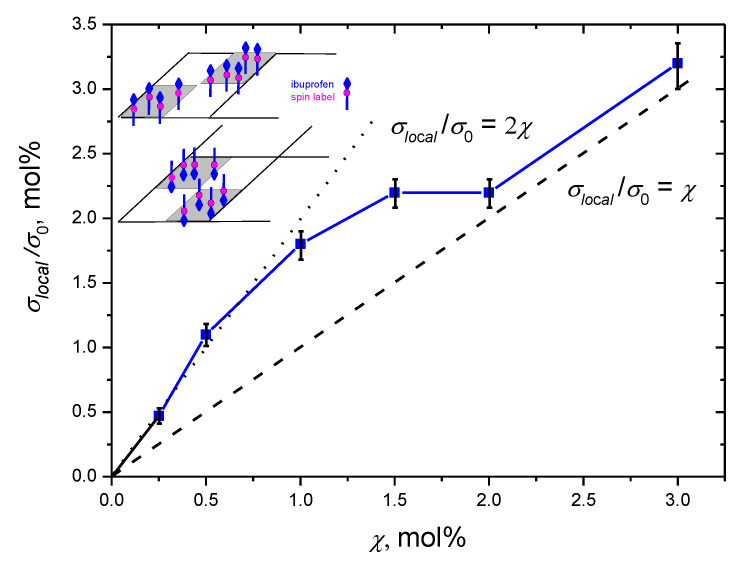
Local ibuprofen-SL surface concentration *σ_local_* in the POPC bilayer as a function of molar concentration *χ*. The data are presented in dimensionless units, *σ_local_*/σ_0_, where *σ*_0_ represents the lipid surface concentration (assessed as 1.7 nm^−2^, see text). The dashed and dotted lines present the functions *σ_local_*/*σ*_0_ = *χ* and *σ_local_*/*σ*_0_ = 2*χ*, respectively. The inset schematically shows a possible distribution pattern of ibuprofen between two leaflets of the bilayer (a chess box model).

Figure 4 presents the *σ_local_* values obtained using these linear approximations and Equation (9). The data are given as a function of the ibuprofen-SL content *χ*, in the dimensionless units, *σ_local_*/*σ*_0_, where *σ*_0_ the surface lipid concentration. The latter was assessed as *~*1/*A*_L_, where *A*_L_ is the area per lipid, which for the POPC bilayers was reported to lie between 0.54 and 0.68 nm^2^ [44]. In our calculations, we use the assessment *σ*_0_ ≈ 1.7 nm^−2^. 

From the data in Figure 4, one may conclude, first, that in all cases *σ_loc_* ≥ *χσ*_0_. Thus, the model of two-dimensional random spin distribution does not contradict the experiment. Then, for *χ* < 1 mol%, *σ_loc_* is twice as large as *χσ*_0_. This enhancement unambiguously demonstrates the heterogeneity of the spatial distribution of the ibuprofen-SL molecules. For *χ* > 2 mol%, Figure 4 shows that an approximate equality of *σ_loc_* and *χσ*_0_ is attained, which means that the spatial distribution of ibuprofen-SL in the bilayer becomes random. 

In our opinion, the revealed concentration dependence can be explained by the following model. At χ < 1 mol%, ibuprofen-SL molecules are predominantly assembled in only one leaflet of the bilayer, forming large clusters that appear alternately in two leaflets. Schematically, this spatial distribution resembles a chess box, as shown in the insert to Figure 4. Then in each leaflet, the local surface concentration *σ_loc_* should indeed become twice as high. For large *χ* > 2 mol%, these clusters are destroyed, and the spatial distribution becomes homogeneous. 

The maximum achievable surface concentration in the assembly is about 2 mol%, which allows assessing the characteristic distance between the ibuprofen-SL molecules in the assembly as $\sqrt{A_{L}/0.02}\approx$ 5 nm. 

Note that the concentration dependence in Figure 4 resembles that seen in the insert to Figure 2a for the CW EPR linewidth at room temperature: the EPR linewidth there also increases sharply for *χ* < 1 mol%, and for larger *χ* the increase becomes slower. Therefore, we can assume that the spatial heterogeneity observed by DEER in frozen samples reflects what happens at physiological temperatures. Of course, CW EPR data, taken alone, cannot serve as a source of information on this heterogeneity because of the smallness of the effect and the possibility of different explanations (dipole-dipole or exchange interaction, slowing down of the motion).

## 5. Conclusions

The DEER study performed here for ibuprofen-SL in a model lipid membrane provided information on the nanoscale spatial distribution of these molecules. The results obtained are consistent with the theory of a two-dimensional random distribution of spins. Therefore, it may be concluded that ibuprofen-SL molecules are located within two planes, for two leaflets of the bilayer. Comparison with theory made it possible to obtain surface concentrations of ibuprofen-SL. For average molar concentrations *χ* less than 1 mol%, these concentrations turned out to be twice as high as *χ*. This increase can be explained by the alternative assembling of ibuprofen-SL in the two opposite leaflets. The maximum achievable surface concentration in the assembly is about 2 mol%; when *χ* is more than 2 mol%, the assembling disappears, and ibuprofen-SL molecules are distributed randomly in each leaflet. 

The data obtained in this study shed new light on the nanoscale spatial arrangement of ibuprofen in the lipid bilayer, which may be useful in the development of theoretical models of ibuprofen’s interaction with the membrane in the future.

## Data Availability

Data is contained within the article.

## Sample Availability

Samples of the synthesized compounds are available from the authors.
